# Supplementary material for: Retention in care and antiretroviral therapy adherence among Medicaid beneficiaries with HIV, 2001–2015
Source: medRxiv. 2024 May 13:2024.05.13.24307278. Preprint. [Version 1] doi: 10.1101/2024.05.13.24307278 (PMC11118595; doi:10.1101/2024.05.13.24307278)
Supplement: Supplement 1 [file media-1.pdf]

Appendix Table of Contents.

Item	Description	Page
Table S1	Codes used to define key variables	3
Table S2	Weighted prevalence of each level of the HIV care cascade and cumulative incidence of mortality by end of the calendar year and the annual increase in the cumulative incidence of mortality by sex, using an adherence level of 80%	4
Figure S1	Weighted prevalence of each level of the HIV care cascade by end of the calendar year not considering incidence of death by sex, using an adherence level of 80%	5
Table S3	Weighted prevalence of each level of the HIV care cascade and cumulative incidence of mortality by end of the calendar year and the annual increase in the cumulative incidence of mortality by race/ethnicity, using an adherence level of 80%	6
Figure S2	Weighted prevalence of each level of the HIV care cascade by end of the calendar year not considering incidence of death by race/ethnicity, using an adherence level of 80%	8
Figure S3	Weighted prevalence of each level of the HIV care cascade and cumulative incidence of mortality by end of the calendar year by US Census Region, using an adherence level of 80%	9
Figure S4	Weighted prevalence of each level of the HIV care cascade by end of the calendar year not considering incidence of death by US Census Region, using an adherence level of 80%	10
Table S4	Weighted prevalence of each level of the HIV care cascade and cumulative incidence of mortality by end of the calendar year, using an adherence level of 0%	11
Table S5	Weighted prevalence of each level of the HIV care cascade and cumulative incidence of mortality by end of the calendar year, using an adherence level of 70%	12
Table S6	Weighted prevalence of each level of the HIV care cascade and cumulative incidence of mortality by end of the calendar year, using an adherence level of 90%	13
Table S7	Crude prevalence of each level of the HIV care cascade and cumulative incidence of mortality by end of the calendar year, using an adherence level of 80%	14

Table S8	Crude prevalence of each level of the HIV care cascade and cumulative incidence of mortality by end of the calendar year, using an adherence level of 0%	15
Table S9	Crude prevalence of each level of the HIV care cascade and cumulative incidence of mortality by end of the calendar year, using an adherence level of 70%	16
Table S10	Crude prevalence of each level of the HIV care cascade and cumulative incidence of mortality by end of the calendar year, using an adherence level of 90%	17
Table S11	Weighted prevalence of each level of the HIV care cascade and cumulative incidence of mortality by end of the calendar year, using an adherence level of 80% and excluding California	18
Table S12	Weighted prevalence of each level of the HIV care cascade and cumulative incidence of mortality by end of the calendar year, using an adherence level of 80% and using September 30, 2015 as the administrative censoring date for all states	19

Table S1. Codes used to define key variables[a]

Variable	CPT/HCPCS	ICD-9
HIV Diagnosis	---	042, 079.53, 795.71, V08
Office visits[b]	99201, 99202, 99203, 99204, 99205, 99212, 99213, 99214, 99215	---
CD4/CD8 measurements	86359, 86360, 86361	---
Viral load tests	87534, 87535, 87536, 87537, 87538, 87539	---
Myocardial infarction	---	410.X, 412.X
Congestive heart failure	---	398.91, 402.01, 402.11, 402.91, 404.01, 404.03, 404.11, 404.13, 404.91, 404.93, 425.4-425.9, 428.X
Peripheral vascular disease	---	093.0, 437.3, 440.X, 441.X, 443.1-443.9, 447.1, 557.1, 557.9, V43.4
Cerebrovascular disease	---	362.34, 430.X-438.X
Dementia	---	290.X, 294.1, 331.2
Chronic pulmonary disease	---	416.8, 416.9, 490.X-505.X, 506.4, 508.1, 508.8
Rheumatic disease	---	446.5, 710.0-710.4, 714.0-714.2, 714.8, 725.X
Peptic ulcer disease	---	531.X-534.X
Mild liver disease	---	070.22, 070.23, 070.32, 070.33, 070.44, 070.54, 070.6, 070.9, 570.X, 571.X, 573.3, 573.4, 573.8, 573.9, V42.7
Moderate/severe liver disease		456.0-456.2, 572.2-572.8
Diabetes without chronic complication	---	250.0-250.3, 250.8, 250.9
Diabetes with chronic complication	---	250.4-250.7
Hemiplegia/paraplegia	---	334.1, 342.X, 343.X, 344.0-344.6, 344.9
Renal disease	---	403.01, 403.11, 403.91, 404.02, 404.03, 404.12, 404.13, 404.92, 404.93, 582.X, 583.0-583.7, 585.X, 586.X, 588.0, V42.0, V45.1, V56.X
Malignancy	---	140.X-172.X, 174.X-195.8, 200.X-208.X, 238.6
Metastatic solid tumor	---	196.X-199.X

Abbreviations: CPT, current procedural terminology; HCPCS, healthcare common procedure coding system; ICD, international classification of diseases

[a]See supplemental file ndc_class.txt for NDC codes used to identify antiretroviral therapy

[b]Where revenue center code not in 0450-0459, 0981, 0516, 0526

Table S2. Weighted prevalence of each level of the HIV care cascade and cumulative incidence of mortality by end of the calendar year and the annual increase in the cumulative incidence of mortality by sex, using an adherence level of 80%

Calendar Year	Not retained Not ART adherent		Retained Not ART adherent		Not retained ART adherent		Retained ART adherent		Mortality Incidence		Annual Increase in Mortality	
	%	95% CI	%	95% CI	%	95% CI	%	95% CI	%	95% CI	%	95% CI
Female beneficiaries												
2002	25.0	(24.5, 25.6)	46.4	(45.9, 47.0)	4.5	(4.2, 4.7)	20.2	(19.8, 20.6)	3.9	(3.7, 4.1)	2.5	(2.3, 2.8)
2003	26.5	(26.0, 27.1)	40.7	(40.2, 41.3)	5.0	(4.8, 5.3)	21.8	(21.4, 22.3)	5.9	(5.7, 6.2)	2.1	(1.7, 2.4)
2004	24.8	(24.3, 25.3)	38.5	(38.0, 39.1)	6.1	(5.8, 6.4)	22.4	(21.9, 22.8)	8.1	(7.8, 8.5)	2.2	(1.8, 2.6)
2005	25.4	(24.9, 25.9)	35.9	(35.3, 36.4)	6.3	(6.0, 6.6)	22.2	(21.8, 22.7)	10.1	(9.8, 10.5)	2.0	(1.5, 2.5)
2006	24.0	(23.5, 24.5)	31.3	(30.7, 31.8)	7.2	(6.9, 7.5)	25.4	(24.9, 25.9)	12.1	(11.7, 12.5)	1.9	(1.4, 2.4)
2007	23.9	(23.4, 24.5)	29.1	(28.5, 29.6)	8.0	(7.8, 8.4)	25.3	(24.8, 25.8)	13.6	(13.3, 14.0)	1.6	(1.0, 2.1)
2008	20.2	(19.7, 20.7)	29.2	(28.6, 29.7)	6.8	(6.5, 7.1)	28.5	(28.0, 29.1)	15.3	(14.9, 15.7)	1.7	(1.1, 2.2)
2009	17.3	(16.8, 17.7)	28.1	(27.5, 28.6)	6.0	(5.7, 6.3)	31.6	(31.0, 32.2)	17.0	(16.6, 17.5)	1.7	(1.1, 2.3)
2010	16.9	(16.4, 17.3)	26.5	(26.0, 27.0)	4.8	(4.6, 5.1)	33.2	(32.6, 33.8)	18.6	(18.1, 19.0)	1.6	(0.9, 2.2)
2011	13.2	(12.8, 13.6)	29.1	(28.5, 29.6)	4.5	(4.2, 4.8)	33.3	(32.7, 33.9)	19.9	(19.5, 20.4)	1.4	(0.7, 2.0)
2012	13.5	(13.0, 13.9)	27.8	(27.3, 28.4)	4.5	(4.3, 4.8)	33.0	(32.4, 33.5)	21.2	(20.7, 21.7)	1.3	(0.6, 1.9)
2013	11.0	(10.6, 11.4)	28.9	(28.4, 29.4)	3.7	(3.5, 3.9)	34.0	(33.4, 34.5)	22.4	(21.9, 22.9)	1.2	(0.5, 1.9)
2014	16.7	(16.1, 17.3)	21.9	(21.2, 22.5)	5.2	(4.9, 5.6)	32.7	(32.0, 33.3)	23.5	(23.0, 24.0)	1.1	(0.4, 1.7)
2015	21.0	(20.3, 21.7)	16.7	(16.2, 17.2)	9.1	(8.7, 9.6)	29.0	(28.4, 29.6)	24.2	(23.8, 24.8)	0.8	(0.1, 1.5)
Male beneficiaries												
2002	24.7	(24.2, 25.1)	40.6	(40.1, 41.1)	6.5	(6.3, 6.8)	23.6	(23.2, 24.0)	4.6	(4.3, 4.8)	3.0	(2.7, 3.3)
2003	24.4	(23.9, 24.9)	36.2	(35.7, 36.7)	6.7	(6.4, 6.9)	25.6	(25.1, 26.0)	7.2	(6.9, 7.5)	2.6	(2.2, 3.0)
2004	23.8	(23.3, 24.3)	32.9	(32.4, 33.3)	7.9	(7.6, 8.2)	25.8	(25.4, 26.3)	9.7	(9.3, 10.0)	2.5	(2.1, 2.9)
2005	23.6	(23.2, 24.1)	30.9	(30.3, 31.3)	7.7	(7.5, 8.0)	25.7	(25.2, 26.2)	12.1	(11.7, 12.5)	2.5	(2.0, 2.9)
2006	21.7	(21.2, 22.1)	26.4	(25.9, 26.8)	8.8	(8.6, 9.2)	28.7	(28.2, 29.2)	14.4	(14.0, 14.8)	2.3	(1.8, 2.8)
2007	22.8	(22.4, 23.3)	22.6	(22.2, 23.0)	9.4	(9.1, 9.7)	29.0	(28.4, 29.4)	16.3	(15.9, 16.7)	1.9	(1.3, 2.4)
2008	19.1	(18.6, 19.6)	22.9	(22.5, 23.4)	8.3	(8.0, 8.6)	31.5	(31.1, 32.0)	18.1	(17.7, 18.6)	1.9	(1.3, 2.5)
2009	16.8	(16.3, 17.2)	22.3	(21.9, 22.8)	7.2	(7.0, 7.5)	33.7	(33.2, 34.2)	20.0	(19.6, 20.5)	1.9	(1.2, 2.5)
2010	17.1	(16.7, 17.5)	20.1	(19.7, 20.5)	6.3	(6.1, 6.6)	34.4	(33.9, 34.9)	22.0	(21.6, 22.5)	2.0	(1.4, 2.7)
2011	12.6	(12.3, 13.0)	22.9	(22.5, 23.4)	6.0	(5.8, 6.3)	34.8	(34.3, 35.3)	23.6	(23.1, 24.1)	1.6	(0.9, 2.2)
2012	12.4	(12.1, 12.8)	22.0	(21.6, 22.4)	5.4	(5.2, 5.6)	35.0	(34.6, 35.6)	25.1	(24.6, 25.6)	1.5	(0.8, 2.2)
2013	10.1	(9.7, 10.4)	21.2	(20.8, 21.6)	5.0	(4.8, 5.2)	37.2	(36.7, 37.7)	26.5	(26.1, 27.0)	1.4	(0.8, 2.1)
2014	11.6	(11.2, 12.0)	17.7	(17.3, 18.1)	6.3	(6.0, 6.6)	37.0	(36.4, 37.5)	27.4	(26.9, 27.9)	0.9	(0.2, 1.6)
2015	14.4	(14.0, 14.8)	14.1	(13.8, 14.4)	10.6	(10.3, 11.0)	32.9	(32.4, 33.3)	28.0	(27.5, 28.5)	0.6	(0.0, 1.3)

Abbreviations: ART, antiretroviral therapy; CI, confidence interval

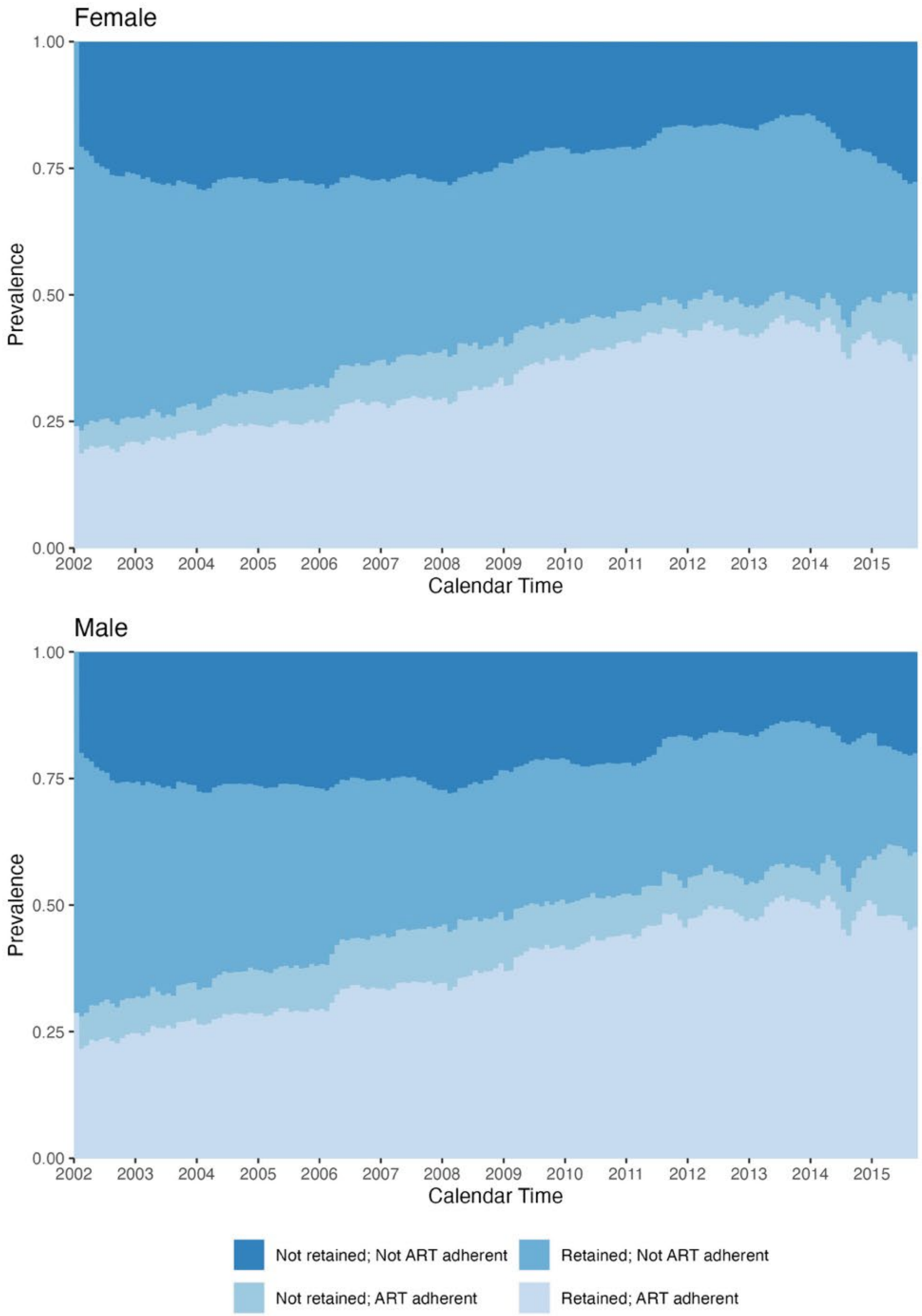

Figure S1. Weighted prevalence of each level of the HIV care cascade by end of the calendar year not considering incidence of death by sex, using an adherence level of 80%

Table S3. Weighted prevalence of each level of the HIV care cascade and cumulative incidence of mortality by end of the calendar year and the annual increase in the cumulative incidence of mortality by race/ethnicity, using an adherence level of 80%

Calendar Year	Not retained Not ART adherent		Retained Not ART adherent		Not retained ART adherent		Retained ART adherent		Mortality Incidence		Annual Increase in Mortality	
	%	95% CI	%	95% CI	%	95% CI	%	95% CI	%	95% CI	%	95% CI
Non-Hispanic Black beneficiaries												
2002	27.6	(27.1, 28.2)	42.4	(41.8, 42.9)	5.6	(5.3, 5.9)	19.4	(19.0, 19.8)	5.0	(4.8, 5.3)	3.3	(3.0, 3.6)
2003	28.4	(27.9, 28.9)	37.4	(36.9, 37.9)	6.1	(5.8, 6.3)	20.5	(20.2, 21.0)	7.6	(7.4, 7.9)	2.6	(2.3, 3.0)
2004	26.3	(25.8, 26.8)	34.7	(34.2, 35.2)	7.3	(7.0, 7.6)	21.1	(20.7, 21.5)	10.5	(10.2, 10.9)	2.8	(2.4, 3.3)
2005	26.6	(26.1, 27.1)	32.4	(31.9, 32.8)	7.3	(7.0, 7.6)	20.7	(20.3, 21.1)	13.0	(12.7, 13.4)	2.6	(2.1, 3.1)
2006	24.6	(24.2, 25.1)	28.1	(27.6, 28.6)	8.2	(7.9, 8.5)	23.6	(23.1, 24.0)	15.5	(15.1, 15.9)	2.5	(1.9, 3.0)
2007	24.4	(23.9, 24.8)	25.3	(24.9, 25.7)	9.0	(8.7, 9.3)	23.7	(23.2, 24.1)	17.6	(17.2, 18.1)	2.1	(1.5, 2.7)
2008	20.2	(19.7, 20.7)	25.7	(25.3, 26.2)	7.9	(7.6, 8.2)	26.5	(26.0, 26.9)	19.7	(19.3, 20.2)	2.1	(1.5, 2.7)
2009	17.3	(16.9, 17.8)	25.0	(24.6, 25.4)	6.7	(6.4, 6.9)	29.3	(28.8, 29.8)	21.7	(21.3, 22.2)	2.0	(1.4, 2.6)
2010	17.5	(17.0, 17.9)	22.7	(22.3, 23.2)	5.7	(5.4, 6.0)	30.4	(30.0, 30.9)	23.7	(23.3, 24.2)	2.0	(1.3, 2.6)
2011	13.9	(13.5, 14.3)	25.5	(25.1, 26.0)	5.5	(5.3, 5.8)	29.8	(29.3, 30.2)	25.3	(24.8, 25.7)	1.5	(0.9, 2.2)
2012	13.7	(13.4, 14.1)	24.0	(23.5, 24.4)	5.2	(5.0, 5.4)	30.4	(29.9, 30.9)	26.7	(26.3, 27.2)	1.4	(0.8, 2.1)
2013	11.3	(10.9, 11.6)	23.3	(22.9, 23.7)	4.5	(4.3, 4.7)	32.9	(32.5, 33.4)	28.0	(27.6, 28.6)	1.4	(0.7, 2.0)
2014	14.4	(13.9, 14.8)	19.6	(19.1, 20.0)	6.0	(5.7, 6.3)	30.9	(30.4, 31.5)	29.2	(28.7, 29.6)	1.1	(0.4, 1.8)
2015	18.3	(17.9, 18.8)	14.6	(14.1, 15.0)	9.9	(9.5, 10.3)	27.4	(26.8, 27.9)	29.8	(29.4, 30.3)	0.7	(0.0, 1.3)
Non-Hispanic white beneficiaries												
2002	19.2	(18.5, 19.9)	47.0	(46.1, 47.8)	4.3	(4.0, 4.7)	25.7	(24.9, 26.4)	3.8	(3.4, 4.1)	2.4	(2.0, 2.8)
2003	19.8	(19.1, 20.5)	41.7	(40.9, 42.5)	5.0	(4.6, 5.4)	27.4	(26.7, 28.2)	6.1	(5.6, 6.4)	2.3	(1.7, 2.8)
2004	20.0	(19.4, 20.7)	37.6	(36.7, 38.4)	5.9	(5.5, 6.3)	28.5	(27.8, 29.2)	8.1	(7.6, 8.5)	2.0	(1.4, 2.6)
2005	19.1	(18.5, 19.8)	35.5	(34.7, 36.3)	5.2	(4.8, 5.6)	29.7	(28.9, 30.4)	10.4	(10.0, 11.0)	2.4	(1.7, 3.1)
2006	17.9	(17.2, 18.6)	31.1	(30.4, 31.9)	6.3	(5.9, 6.7)	32.1	(31.3, 32.9)	12.6	(12.0, 13.2)	2.2	(1.4, 2.9)
2007	21.5	(20.7, 22.3)	25.7	(24.9, 26.5)	6.0	(5.6, 6.4)	32.7	(31.8, 33.5)	14.1	(13.5, 14.7)	1.5	(0.7, 2.3)
2008	19.0	(18.3, 19.8)	25.3	(24.6, 26.1)	5.5	(5.1, 5.9)	34.4	(33.6, 35.2)	15.7	(15.2, 16.4)	1.6	(0.8, 2.5)
2009	17.3	(16.5, 18.2)	24.2	(23.4, 25.1)	4.7	(4.3, 5.1)	36.2	(35.4, 37.0)	17.4	(16.8, 18.0)	1.7	(0.8, 2.5)
2010	18.0	(17.2, 18.7)	21.4	(20.7, 22.2)	5.1	(4.7, 5.5)	36.2	(35.3, 37.0)	19.3	(18.7, 20.0)	1.9	(1.0, 2.8)
2011	11.1	(10.5, 11.8)	23.1	(22.3, 23.8)	5.0	(4.6, 5.4)	40.1	(39.2, 40.9)	20.7	(20.0, 21.5)	1.4	(0.4, 2.4)
2012	11.6	(10.9, 12.2)	24.0	(23.3, 24.8)	4.7	(4.3, 5.1)	37.5	(36.5, 38.3)	22.3	(21.5, 23.0)	1.5	(0.5, 2.6)
2013	9.5	(8.9, 10.1)	24.5	(23.7, 25.3)	4.0	(3.6, 4.3)	38.4	(37.6, 39.2)	23.7	(23.0, 24.5)	1.4	(0.4, 2.5)
2014	12.7	(12.1, 13.5)	18.0	(17.4, 18.7)	4.9	(4.4, 5.3)	40.0	(39.0, 40.7)	24.4	(23.7, 25.2)	0.7	(0.0, 1.8)
2015	15.1	(14.4, 15.8)	14.8	(14.3, 15.5)	9.4	(8.8, 9.9)	35.7	(34.8, 36.4)	25.0	(24.3, 25.8)	0.6	(0.0, 1.6)
Hispanic beneficiaries												
2002	23.3	(22.3, 24.2)	41.7	(40.6, 42.8)	6.7	(6.1, 7.3)	24.2	(23.1, 25.2)	4.2	(3.7, 4.7)	3.0	(2.4, 3.6)
2003	22.9	(21.8, 23.9)	37.1	(35.8, 38.2)	6.7	(6.0, 7.4)	27.0	(25.9, 28.2)	6.3	(5.8, 7.0)	2.1	(1.4, 2.9)
2004	22.6	(21.5, 23.8)	33.5	(32.4, 34.8)	7.4	(6.7, 8.0)	28.2	(27.1, 29.3)	8.3	(7.7, 9.0)	2.0	(1.1, 2.9)
2005	21.4	(20.2, 22.5)	33.8	(32.6, 35.1)	6.8	(6.2, 7.5)	27.6	(26.4, 28.7)	10.4	(9.7, 11.1)	2.1	(1.1, 3.0)
2006	19.1	(17.9, 20.3)	27.9	(26.6, 29.1)	7.4	(6.7, 8.1)	32.9	(31.7, 34.1)	12.7	(11.9, 13.5)	2.3	(1.2, 3.4)

2007	20.2	(19.0, 21.4)	25.3	(24.2, 26.5)	8.2	(7.4, 8.9)	32.7	(31.4, 33.9)	13.7	(12.8, 14.5)	1.1	(0.0, 2.2)
2008	17.4	(16.1, 18.4)	24.4	(23.3, 25.6)	7.3	(6.6, 8.1)	35.8	(34.6, 37.2)	15.0	(14.2, 15.9)	1.3	(0.1, 2.5)
2009	14.2	(13.1, 15.3)	24.2	(23.1, 25.5)	7.2	(6.4, 8.0)	37.5	(36.2, 38.8)	16.9	(15.9, 17.8)	1.8	(0.5, 3.1)
2010	14.1	(13.1, 15.2)	23.3	(22.2, 24.4)	6.9	(6.1, 7.6)	37.6	(36.2, 38.8)	18.2	(17.2, 19.2)	1.3	(0.0, 2.7)
2011	12.5	(11.6, 13.5)	22.4	(21.4, 23.6)	6.8	(6.1, 7.6)	38.8	(37.4, 40.0)	19.5	(18.5, 20.6)	1.3	(0.0, 2.8)
2012	13.4	(12.5, 14.4)	21.5	(20.3, 22.6)	7.2	(6.4, 7.8)	37.2	(35.8, 38.7)	20.7	(19.7, 21.8)	1.3	(0.0, 2.7)
2013	10.1	(9.2, 11.2)	21.4	(20.2, 22.5)	6.9	(6.2, 7.6)	39.7	(38.4, 41.0)	21.9	(20.9, 23.0)	1.2	(0.0, 2.7)
2014	13.4	(12.4, 14.5)	17.0	(16.1, 18.0)	8.1	(7.2, 8.9)	38.9	(37.6, 40.1)	22.6	(21.6, 23.7)	0.6	(0.0, 2.1)
2015	15.1	(14.0, 16.1)	13.5	(12.8, 14.3)	12.0	(11.2, 12.9)	36.4	(35.2, 37.5)	23.0	(22.0, 24.2)	0.5	(0.0, 2.0)

Abbreviations: ART, antiretroviral therapy; CI, confidence interval

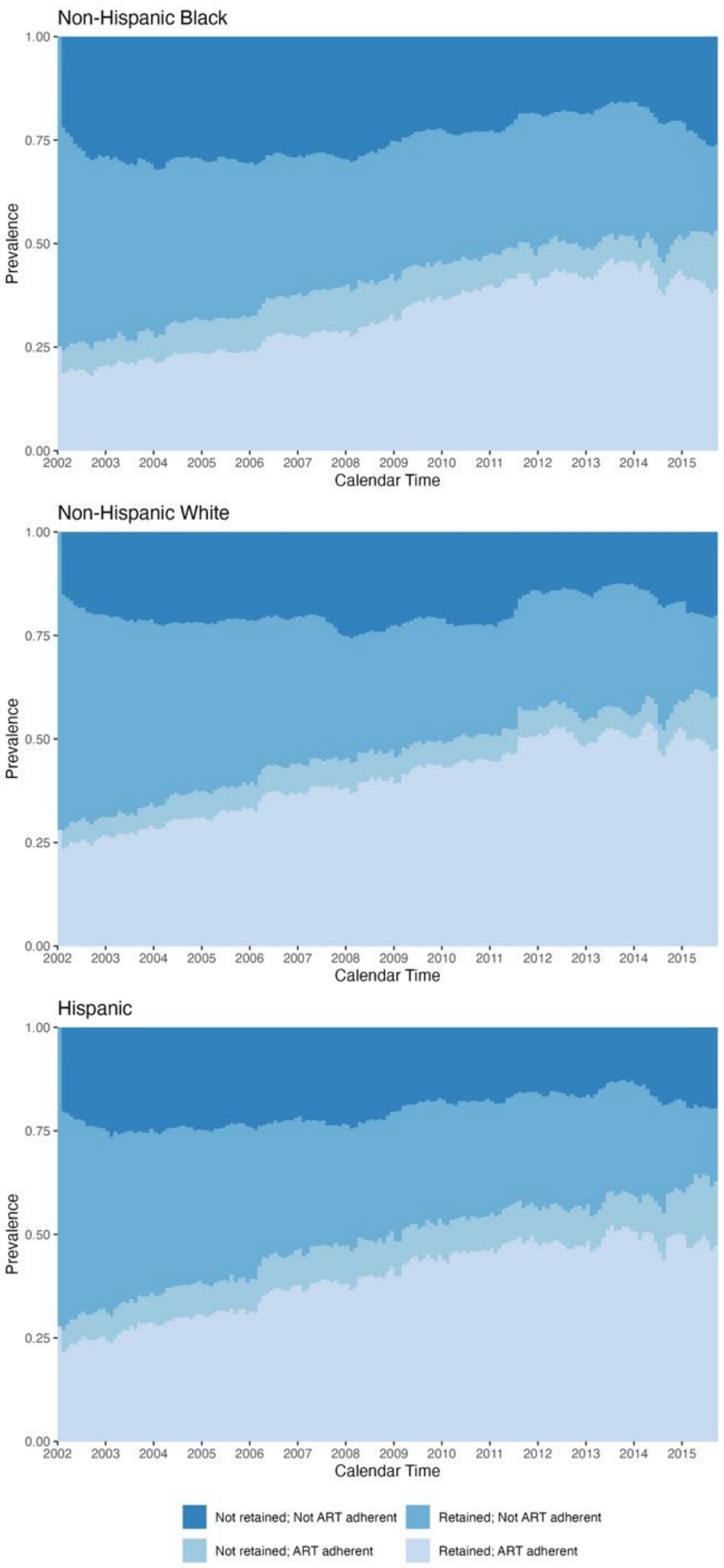

Figure S2. Weighted prevalence of each level of the HIV care cascade by end of the calendar year not considering incidence of death by race/ethnicity, using an adherence level of 80%

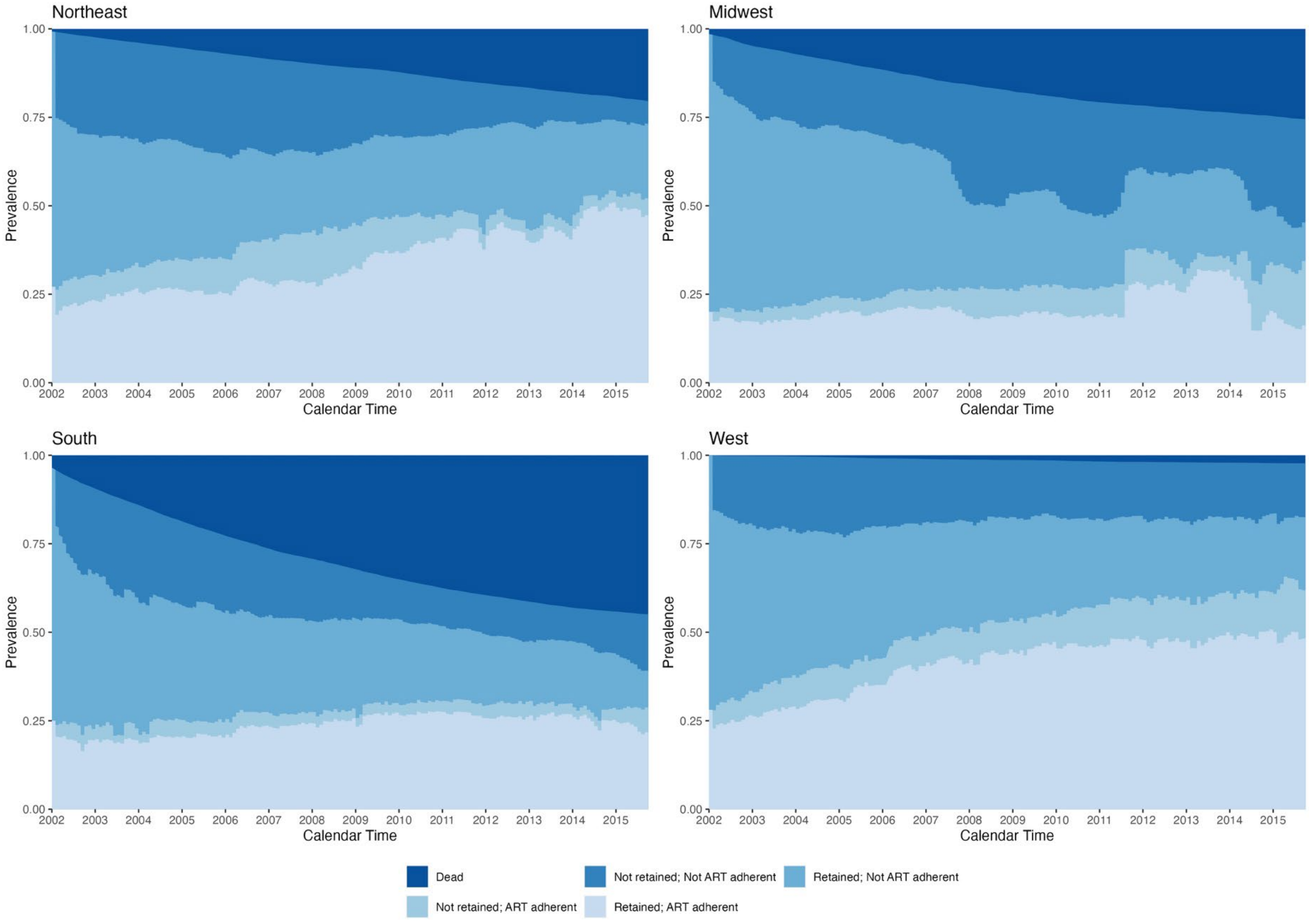

Figure S3. Weighted prevalence of each level of the HIV care cascade and cumulative incidence of mortality by end of the calendar year by US Census Region, using an adherence level of 80%

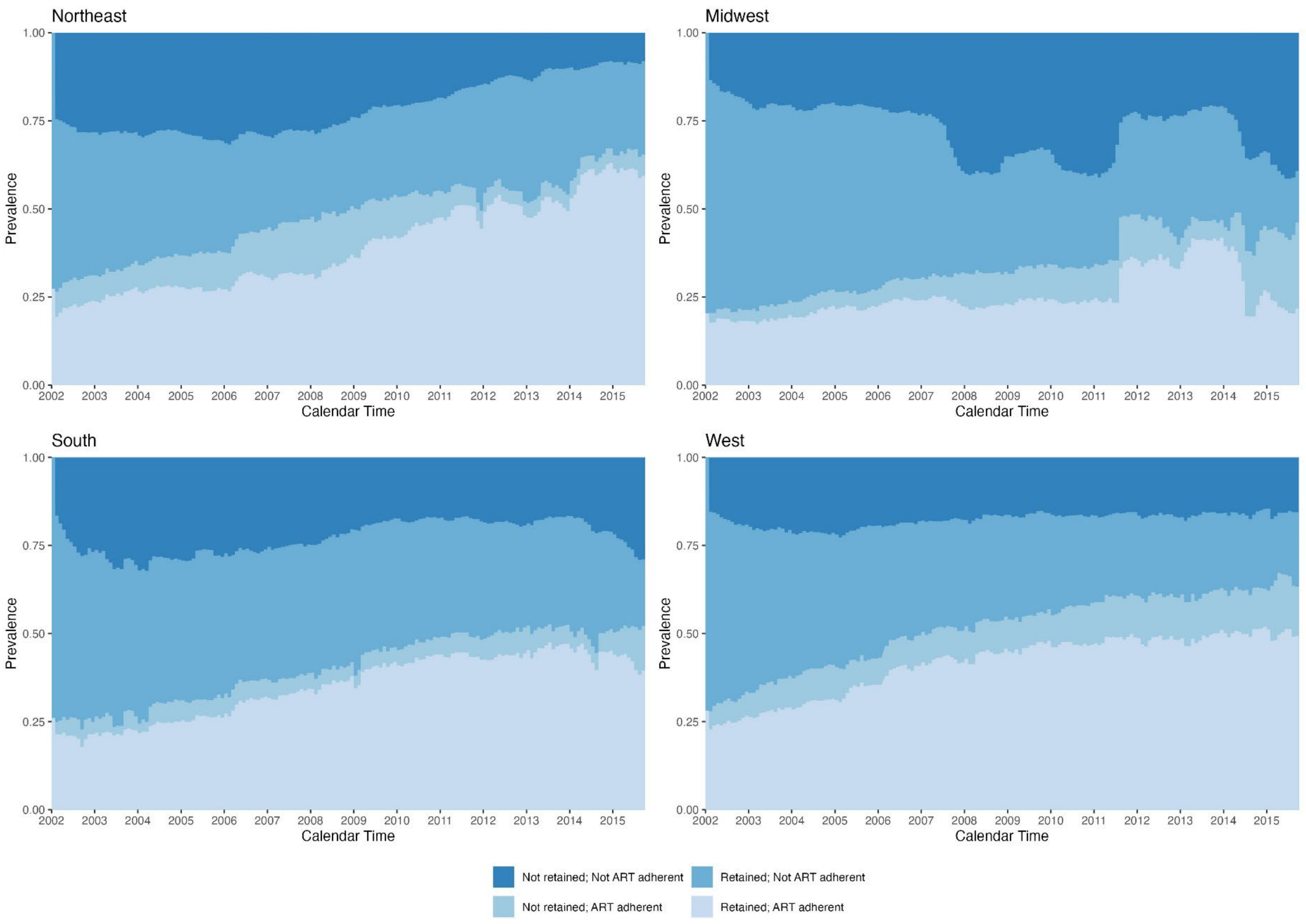

Figure S4. Weighted prevalence of each level of the HIV care cascade by end of the calendar year not considering incidence of death by US Census Region, using an adherence level of 80%

Table S4. Weighted prevalence of each level of the HIV care cascade and cumulative incidence of mortality by end of the calendar year, using an adherence level of 0%

Calendar Year	Not retained Not ART adherent		Retained Not ART adherent		Not retained ART adherent		Retained ART adherent		Mortality Incidence	
	%	95% CI	%	95% CI	%	95% CI	%	95% CI	%	95% CI
2002	21.9	(21.6, 22.3)	34.4	(34.1, 34.8)	8.4	(8.2, 8.6)	31.0	(30.6, 31.4)	4.3	(4.1, 4.4)
2003	22.7	(22.3, 23.0)	30.1	(29.7, 30.4)	8.5	(8.3, 8.8)	32.2	(31.8, 32.5)	6.6	(6.4, 6.8)
2004	21.7	(21.4, 22.0)	27.5	(27.2, 27.9)	9.6	(9.3, 9.8)	32.2	(31.8, 32.5)	9.0	(8.7, 9.2)
2005	21.9	(21.5, 22.2)	25.5	(25.2, 25.8)	9.7	(9.5, 9.9)	31.7	(31.4, 32.1)	11.2	(11.0, 11.5)
2006	20.0	(19.7, 20.4)	20.5	(20.3, 20.9)	10.9	(10.6, 11.1)	35.2	(34.8, 35.6)	13.4	(13.1, 13.6)
2007	20.8	(20.4, 21.1)	17.7	(17.4, 17.9)	11.4	(11.2, 11.7)	35.0	(34.7, 35.4)	15.1	(14.9, 15.4)
2008	17.6	(17.3, 18.0)	17.9	(17.5, 18.2)	9.7	(9.5, 9.9)	37.9	(37.5, 38.3)	16.9	(16.6, 17.2)
2009	15.3	(15.0, 15.6)	16.7	(16.4, 17.0)	8.6	(8.4, 8.8)	40.7	(40.3, 41.1)	18.7	(18.4, 19.0)
2010	15.4	(15.0, 15.7)	14.3	(14.0, 14.6)	7.5	(7.3, 7.7)	42.3	(41.9, 42.7)	20.6	(20.2, 20.9)
2011	11.3	(11.0, 11.5)	15.8	(15.5, 16.1)	7.1	(6.9, 7.3)	43.8	(43.4, 44.2)	22.0	(21.7, 22.4)
2012	10.8	(10.6, 11.0)	13.8	(13.6, 14.1)	7.1	(6.9, 7.4)	44.8	(44.4, 45.2)	23.5	(23.1, 23.8)
2013	8.9	(8.6, 9.1)	14.9	(14.6, 15.1)	6.0	(5.8, 6.1)	45.5	(45.1, 45.9)	24.8	(24.5, 25.2)
2014	12.0	(11.7, 12.3)	11.5	(11.2, 11.8)	7.7	(7.5, 8.0)	43.0	(42.5, 43.5)	25.7	(25.4, 26.1)
2015	14.2	(13.9, 14.5)	8.2	(8.0, 8.5)	13.2	(12.9, 13.5)	38.0	(37.5, 38.4)	26.4	(26.0, 26.8)

Abbreviations: ART, antiretroviral therapy; CI, confidence interval

Table S5. Weighted prevalence of each level of the HIV care cascade and cumulative incidence of mortality by end of the calendar year, using an adherence level of 70%

Calendar Year	Not retained Not ART adherent		Retained Not ART adherent		Not retained ART adherent		Retained ART adherent		Mortality Incidence	
	%	95% CI	%	95% CI	%	95% CI	%	95% CI	%	95% CI
2002	24.3	(24.0, 24.6)	41.4	(41.1, 41.8)	6.1	(5.9, 6.3)	23.9	(23.6, 24.3)	4.3	(4.1, 4.4)
2003	24.9	(24.5, 25.2)	36.4	(36.0, 36.8)	6.4	(6.2, 6.6)	25.7	(25.4, 26.0)	6.6	(6.4, 6.8)
2004	23.6	(23.3, 24.0)	33.3	(33.0, 33.7)	7.7	(7.5, 7.9)	26.4	(26.0, 26.7)	9.0	(8.8, 9.2)
2005	23.8	(23.4, 24.1)	31.0	(30.7, 31.4)	7.8	(7.5, 8.0)	26.2	(25.9, 26.6)	11.2	(11.0, 11.5)
2006	21.9	(21.6, 22.3)	26.2	(25.9, 26.6)	8.9	(8.7, 9.1)	29.6	(29.2, 29.9)	13.4	(13.1, 13.7)
2007	22.6	(22.2, 22.9)	23.2	(22.8, 23.5)	9.6	(9.3, 9.8)	29.6	(29.2, 29.9)	15.1	(14.9, 15.4)
2008	19.1	(18.8, 19.5)	23.7	(23.4, 24.1)	8.1	(7.9, 8.3)	32.1	(31.8, 32.5)	16.9	(16.6, 17.2)
2009	16.6	(16.2, 16.9)	22.7	(22.4, 23.1)	7.2	(7.0, 7.4)	34.8	(34.4, 35.2)	18.7	(18.5, 19.1)
2010	16.6	(16.3, 16.9)	20.3	(20.0, 20.7)	6.2	(6.0, 6.4)	36.3	(35.9, 36.7)	20.6	(20.3, 20.9)
2011	12.4	(12.1, 12.7)	22.9	(22.6, 23.2)	5.9	(5.7, 6.1)	36.7	(36.3, 37.1)	22.1	(21.8, 22.4)
2012	12.4	(12.1, 12.7)	21.8	(21.4, 22.1)	5.5	(5.4, 5.8)	36.8	(36.4, 37.2)	23.5	(23.2, 23.9)
2013	10.1	(9.9, 10.4)	22.1	(21.8, 22.4)	4.8	(4.7, 5.0)	38.1	(37.7, 38.5)	24.8	(24.5, 25.2)
2014	13.1	(12.8, 13.5)	17.1	(16.8, 17.5)	6.5	(6.3, 6.7)	37.5	(37.1, 38.0)	25.8	(25.4, 26.2)
2015	16.1	(15.7, 16.4)	13.4	(13.1, 13.7)	10.9	(10.6, 11.2)	33.2	(32.7, 33.6)	26.4	(26.1, 26.8)

Abbreviations: ART, antiretroviral therapy; CI, confidence interval

Table S6. Weighted prevalence of each level of the HIV care cascade and cumulative incidence of mortality by end of the calendar year, using an adherence level of 90%

Calendar Year	Not retained Not ART adherent		Retained Not ART adherent		Not retained ART adherent		Retained ART adherent		Mortality Incidence	
	%	95% CI	%	95% CI	%	95% CI	%	95% CI	%	95% CI
2002	25.7	(25.4, 26.1)	46.6	(46.2, 47.0)	4.7	(4.5, 4.9)	18.7	(18.4, 19.0)	4.3	(4.1, 4.4)
2003	26.3	(26.0, 26.7)	41.7	(41.3, 42.1)	5.0	(4.8, 5.2)	20.3	(20.0, 20.6)	6.6	(6.4, 6.8)
2004	25.2	(24.8, 25.5)	38.7	(38.3, 39.1)	6.1	(5.9, 6.3)	21.0	(20.7, 21.3)	9.0	(8.8, 9.2)
2005	25.4	(25.0, 25.7)	36.3	(35.9, 36.7)	6.1	(5.9, 6.3)	20.9	(20.7, 21.3)	11.3	(11.0, 11.5)
2006	23.7	(23.4, 24.1)	32.0	(31.6, 32.3)	7.1	(6.9, 7.3)	23.8	(23.5, 24.2)	13.4	(13.1, 13.7)
2007	24.4	(24.0, 24.7)	28.9	(28.6, 29.3)	7.7	(7.5, 7.9)	23.8	(23.5, 24.2)	15.1	(14.9, 15.4)
2008	20.5	(20.2, 20.8)	29.4	(29.0, 29.7)	6.7	(6.5, 6.9)	26.5	(26.1, 26.9)	16.9	(16.7, 17.3)
2009	17.8	(17.5, 18.1)	28.7	(28.4, 29.1)	5.9	(5.8, 6.1)	28.8	(28.4, 29.1)	18.7	(18.5, 19.1)
2010	17.8	(17.4, 18.1)	26.7	(26.3, 27.1)	5.0	(4.8, 5.2)	30.0	(29.6, 30.3)	20.6	(20.3, 20.9)
2011	13.5	(13.2, 13.8)	29.5	(29.1, 29.8)	4.8	(4.6, 4.9)	30.1	(29.8, 30.5)	22.1	(21.8, 22.4)
2012	13.5	(13.3, 13.8)	28.8	(28.5, 29.2)	4.4	(4.3, 4.6)	29.7	(29.3, 30.0)	23.5	(23.2, 23.9)
2013	11.0	(10.7, 11.3)	28.7	(28.3, 29.0)	4.0	(3.8, 4.1)	31.5	(31.1, 31.9)	24.9	(24.5, 25.2)
2014	14.2	(13.9, 14.6)	23.3	(22.9, 23.7)	5.2	(5.0, 5.5)	31.5	(31.1, 31.9)	25.8	(25.5, 26.2)
2015	18.1	(17.7, 18.4)	18.9	(18.6, 19.3)	8.8	(8.6, 9.1)	27.7	(27.4, 28.1)	26.4	(26.1, 26.8)

Abbreviations: ART, antiretroviral therapy; CI, confidence interval

Table S7. Crude prevalence of each level of the HIV care cascade and cumulative incidence of mortality by end of the calendar, using an adherence level of 80%

Calendar Year	Not retained Not ART adherent		Retained Not ART adherent		Not retained ART adherent		Retained ART adherent		Mortality Incidence	
	%	95% CI	%	95% CI	%	95% CI	%	95% CI	%	95% CI
2002	24.9	(24.5, 25.2)	42.9	(42.5, 43.3)	5.7	(5.5, 5.9)	22.4	(22.0, 22.7)	4.2	(4.0, 4.3)
2003	25.3	(25.0, 25.7)	37.7	(37.3, 38.0)	6.1	(5.9, 6.3)	24.5	(24.2, 24.8)	6.5	(6.3, 6.6)
2004	24.1	(23.8, 24.5)	34.8	(34.4, 35.1)	7.3	(7.1, 7.5)	25.0	(24.7, 25.3)	8.8	(8.5, 9.0)
2005	24.5	(24.1, 24.8)	32.2	(31.9, 32.6)	7.5	(7.3, 7.7)	24.8	(24.5, 25.2)	11.0	(10.7, 11.2)
2006	22.7	(22.4, 23.0)	27.4	(27.1, 27.8)	8.7	(8.5, 8.9)	28.2	(27.8, 28.5)	13.0	(12.8, 13.3)
2007	22.2	(21.9, 22.6)	25.1	(24.7, 25.4)	9.6	(9.3, 9.8)	28.4	(28.0, 28.7)	14.7	(14.5, 15.0)
2008	18.5	(18.2, 18.8)	24.9	(24.6, 25.2)	8.4	(8.2, 8.7)	31.7	(31.3, 32.1)	16.5	(16.2, 16.8)
2009	15.7	(15.4, 16.0)	24.1	(23.8, 24.4)	7.4	(7.2, 7.6)	34.5	(34.2, 34.9)	18.2	(17.9, 18.5)
2010	15.1	(14.8, 15.3)	22.7	(22.4, 23.0)	6.0	(5.8, 6.2)	36.1	(35.8, 36.5)	20.1	(19.8, 20.4)
2011	12.5	(12.2, 12.8)	25.3	(25.0, 25.7)	5.5	(5.3, 5.7)	35.0	(34.7, 35.4)	21.6	(21.3, 22.0)
2012	12.3	(12.0, 12.5)	23.8	(23.5, 24.1)	5.1	(4.9, 5.3)	35.8	(35.4, 36.1)	23.1	(22.7, 23.4)
2013	9.9	(9.7, 10.1)	24.2	(23.9, 24.5)	4.5	(4.3, 4.6)	36.9	(36.6, 37.3)	24.5	(24.1, 24.8)
2014	13.6	(13.3, 14.0)	18.5	(18.2, 18.8)	6.3	(6.1, 6.5)	36.1	(35.7, 36.5)	25.4	(25.1, 25.8)
2015	16.6	(16.3, 17.0)	14.8	(14.5, 15.1)	10.3	(10.0, 10.6)	32.2	(31.8, 32.6)	26.1	(25.7, 26.4)

Abbreviations: ART, antiretroviral therapy; CI, confidence interval

Table S8. Crude prevalence of each level of the HIV care cascade and cumulative incidence of mortality by end of the calendar year, using an adherence level of 0%

Calendar Year	Not retained Not ART adherent		Retained Not ART adherent		Not retained ART adherent		Retained ART adherent		Mortality Incidence	
	%	95% CI	%	95% CI	%	95% CI	%	95% CI	%	95% CI
2002	22.0	(21.6, 22.3)	34.1	(33.7, 34.5)	8.5	(8.3, 8.7)	31.2	(30.9, 31.6)	4.2	(4.0, 4.3)
2003	22.6	(22.3, 23.0)	29.5	(29.2, 29.9)	8.7	(8.4, 8.9)	32.7	(32.4, 33.1)	6.4	(6.2, 6.6)
2004	21.7	(21.3, 22.0)	26.9	(26.6, 27.3)	9.8	(9.6, 10.1)	32.9	(32.5, 33.2)	8.7	(8.5, 9.0)
2005	21.9	(21.6, 22.3)	24.7	(24.3, 25.0)	10.1	(9.8, 10.3)	32.4	(32.0, 32.7)	10.9	(10.7, 11.2)
2006	20.0	(19.7, 20.3)	19.5	(19.2, 19.8)	11.5	(11.2, 11.7)	36.0	(35.6, 36.3)	13.0	(12.8, 13.3)
2007	19.6	(19.3, 20.0)	17.5	(17.2, 17.8)	12.3	(12.0, 12.6)	35.9	(35.5, 36.2)	14.7	(14.4, 15.0)
2008	16.6	(16.2, 16.9)	17.3	(17.0, 17.6)	10.5	(10.3, 10.8)	39.2	(38.8, 39.6)	16.5	(16.2, 16.7)
2009	14.0	(13.7, 14.3)	16.2	(15.9, 16.4)	9.3	(9.0, 9.5)	42.3	(41.9, 42.7)	18.2	(17.9, 18.5)
2010	13.4	(13.1, 13.7)	14.3	(14.0, 14.5)	7.8	(7.6, 8.0)	44.5	(44.1, 44.9)	20.1	(19.8, 20.4)
2011	11.0	(10.7, 11.2)	15.9	(15.6, 16.1)	7.1	(6.9, 7.3)	44.4	(44.0, 44.8)	21.6	(21.3, 21.9)
2012	10.3	(10.1, 10.6)	13.6	(13.4, 13.9)	7.0	(6.8, 7.2)	46.0	(45.6, 46.4)	23.0	(22.7, 23.4)
2013	8.4	(8.2, 8.6)	14.8	(14.6, 15.1)	5.8	(5.6, 6.0)	46.5	(46.2, 46.9)	24.4	(24.1, 24.8)
2014	12.0	(11.7, 12.3)	10.7	(10.4, 10.9)	8.1	(7.8, 8.3)	43.9	(43.4, 44.3)	25.4	(25.1, 25.8)
2015	14.1	(13.8, 14.4)	8.1	(7.9, 8.3)	13.1	(12.8, 13.4)	38.7	(38.3, 39.1)	26.0	(25.7, 26.4)

Abbreviations: ART, antiretroviral therapy; CI, confidence interval

Table S9. Crude prevalence of each level of the HIV care cascade and cumulative incidence of mortality by end of the calendar year, using an adherence level of 70%

Calendar Year	Not retained Not ART adherent		Retained Not ART adherent		Not retained ART adherent		Retained ART adherent		Mortality Incidence	
	%	95% CI	%	95% CI	%	95% CI	%	95% CI	%	95% CI
2002	24.4	(24.0, 24.7)	41.1	(40.7, 41.5)	6.2	(6.0, 6.4)	24.2	(23.9, 24.5)	4.2	(4.0, 4.3)
2003	24.8	(24.5, 25.2)	35.9	(35.5, 36.3)	6.5	(6.3, 6.7)	26.3	(25.9, 26.6)	6.4	(6.3, 6.6)
2004	23.5	(23.2, 23.9)	32.8	(32.4, 33.1)	7.9	(7.7, 8.1)	27.0	(26.7, 27.4)	8.8	(8.5, 9.0)
2005	23.8	(23.5, 24.2)	30.3	(29.9, 30.6)	8.1	(7.9, 8.3)	26.8	(26.5, 27.2)	11.0	(10.7, 11.2)
2006	22.0	(21.6, 22.3)	25.2	(24.9, 25.5)	9.4	(9.2, 9.7)	30.4	(30.1, 30.8)	13.0	(12.8, 13.3)
2007	21.5	(21.1, 21.8)	23.0	(22.6, 23.3)	10.4	(10.1, 10.6)	30.5	(30.1, 30.8)	14.7	(14.4, 15.0)
2008	18.1	(17.7, 18.4)	23.2	(22.8, 23.5)	8.9	(8.6, 9.1)	33.4	(33.1, 33.8)	16.5	(16.2, 16.8)
2009	15.3	(15.0, 15.6)	22.2	(21.9, 22.5)	7.9	(7.6, 8.1)	36.4	(36.0, 36.8)	18.2	(17.9, 18.5)
2010	14.6	(14.3, 14.9)	20.3	(20.0, 20.6)	6.5	(6.3, 6.7)	38.5	(38.1, 38.9)	20.1	(19.8, 20.4)
2011	12.1	(11.8, 12.3)	23.0	(22.7, 23.3)	5.9	(5.7, 6.1)	37.4	(37.0, 37.7)	21.6	(21.3, 21.9)
2012	11.8	(11.6, 12.1)	21.4	(21.1, 21.7)	5.5	(5.3, 5.7)	38.2	(37.9, 38.6)	23.1	(22.7, 23.4)
2013	9.6	(9.4, 9.8)	22.0	(21.8, 22.3)	4.8	(4.6, 4.9)	39.1	(38.8, 39.5)	24.4	(24.1, 24.8)
2014	13.2	(12.9, 13.5)	16.6	(16.3, 16.9)	6.8	(6.5, 7.0)	38.0	(37.6, 38.4)	25.4	(25.1, 25.8)
2015	16.1	(15.7, 16.4)	13.3	(13.1, 13.6)	10.9	(10.6, 11.2)	33.6	(33.2, 34.0)	26.1	(25.7, 26.4)

Abbreviations: ART, antiretroviral therapy; CI, confidence interval

Table S10. Crude prevalence of each level of the HIV care cascade and cumulative incidence of mortality by end of the calendar, using an adherence level of 90%

Calendar Year	Not retained Not ART adherent		Retained Not ART adherent		Not retained ART adherent		Retained ART adherent		Mortality Incidence	
	%	95% CI	%	95% CI	%	95% CI	%	95% CI	%	95% CI
2002	25.8	(25.4, 26.1)	46.3	(45.9, 46.7)	4.8	(4.6, 5.0)	19.0	(18.7, 19.3)	4.2	(4.0, 4.3)
2003	26.3	(25.9, 26.7)	41.3	(40.9, 41.7)	5.1	(4.9, 5.3)	20.8	(20.5, 21.1)	6.5	(6.3, 6.7)
2004	25.1	(24.8, 25.5)	38.2	(37.8, 38.6)	6.3	(6.1, 6.5)	21.6	(21.3, 21.9)	8.8	(8.5, 9.0)
2005	25.5	(25.2, 25.9)	35.6	(35.2, 36.0)	6.4	(6.2, 6.6)	21.5	(21.2, 21.8)	11.0	(10.7, 11.2)
2006	23.8	(23.5, 24.2)	31.0	(30.7, 31.4)	7.5	(7.3, 7.7)	24.6	(24.2, 24.9)	13.0	(12.8, 13.3)
2007	23.4	(23.1, 23.7)	28.8	(28.4, 29.1)	8.4	(8.2, 8.6)	24.7	(24.4, 25.0)	14.7	(14.5, 15.0)
2008	19.5	(19.2, 19.9)	28.9	(28.5, 29.2)	7.4	(7.2, 7.6)	27.7	(27.4, 28.1)	16.5	(16.2, 16.8)
2009	16.6	(16.3, 16.9)	28.3	(28.0, 28.7)	6.5	(6.3, 6.7)	30.3	(29.9, 30.7)	18.2	(17.9, 18.5)
2010	15.8	(15.5, 16.1)	26.9	(26.5, 27.2)	5.3	(5.1, 5.4)	32.0	(31.6, 32.3)	20.1	(19.8, 20.4)
2011	13.2	(12.9, 13.4)	29.5	(29.2, 29.8)	4.8	(4.7, 5.0)	30.9	(30.5, 31.2)	21.6	(21.3, 22.0)
2012	13.0	(12.7, 13.2)	28.5	(28.1, 28.8)	4.4	(4.2, 4.6)	31.1	(30.8, 31.5)	23.1	(22.7, 23.4)
2013	10.4	(10.2, 10.7)	28.6	(28.3, 29.0)	4.0	(3.8, 4.1)	32.5	(32.1, 32.8)	24.5	(24.1, 24.8)
2014	14.4	(14.1, 14.7)	22.8	(22.5, 23.2)	5.5	(5.3, 5.7)	31.8	(31.4, 32.2)	25.5	(25.1, 25.8)
2015	18.0	(17.7, 18.4)	18.9	(18.6, 19.2)	8.8	(8.6, 9.1)	28.2	(27.8, 28.5)	26.1	(25.7, 26.4)

Abbreviations: ART, antiretroviral therapy; CI, confidence interval

Table S11. Weighted prevalence of each level of the HIV care cascade and cumulative incidence of mortality by end of the calendar year, using an adherence level of 80% and excluding California

Calendar Year	Not retained Not ART adherent		Retained Not ART adherent		Not retained ART adherent		Retained ART adherent		Mortality Incidence	
	%	95% CI	%	95% CI	%	95% CI	%	95% CI	%	95% CI
2002	25.6	(25.3, 26.0)	42.7	(42.3, 43.1)	5.4	(5.2, 5.6)	21.5	(21.1, 21.8)	4.8	(4.6, 5.0)
2003	26.0	(25.7, 26.4)	37.8	(37.4, 38.1)	5.5	(5.3, 5.7)	23.3	(22.9, 23.6)	7.5	(7.3, 7.7)
2004	24.6	(24.3, 24.9)	35.0	(34.6, 35.4)	6.7	(6.5, 6.9)	23.5	(23.1, 23.9)	10.2	(9.9, 10.5)
2005	25.1	(24.7, 25.4)	32.4	(32.0, 32.7)	7.1	(6.9, 7.3)	22.7	(22.4, 23.1)	12.7	(12.5, 13.0)
2006	23.3	(23.0, 23.6)	27.9	(27.6, 28.3)	8.1	(7.9, 8.3)	25.5	(25.2, 25.8)	15.2	(14.9, 15.5)
2007	24.1	(23.8, 24.4)	24.4	(24.1, 24.8)	8.8	(8.5, 9.0)	25.6	(25.2, 25.9)	17.1	(16.8, 17.5)
2008	20.0	(19.7, 20.4)	25.0	(24.6, 25.3)	7.5	(7.3, 7.7)	28.4	(28.0, 28.7)	19.2	(18.8, 19.5)
2009	17.2	(16.9, 17.6)	24.2	(23.9, 24.6)	6.4	(6.2, 6.6)	30.9	(30.6, 31.3)	21.2	(20.9, 21.6)
2010	17.1	(16.8, 17.5)	22.4	(22.0, 22.7)	4.8	(4.6, 5.0)	32.4	(32.0, 32.8)	23.3	(22.9, 23.7)
2011	12.5	(12.2, 12.8)	25.6	(25.2, 26.0)	4.5	(4.3, 4.7)	32.5	(32.0, 32.9)	25.0	(24.6, 25.3)
2012	12.3	(12.0, 12.6)	24.4	(24.1, 24.8)	4.2	(4.0, 4.3)	32.5	(32.1, 32.9)	26.5	(26.2, 26.9)
2013	9.7	(9.5, 10.0)	24.5	(24.2, 24.8)	3.6	(3.4, 3.7)	34.2	(33.8, 34.5)	28.0	(27.7, 28.4)
2014	14.0	(13.6, 14.4)	18.7	(18.4, 19.1)	5.5	(5.2, 5.7)	32.6	(32.1, 33.1)	29.2	(28.8, 29.6)
2015	18.5	(18.1, 19.0)	14.1	(13.8, 14.5)	9.9	(9.6, 10.3)	27.3	(26.9, 27.8)	30.1	(29.7, 30.5)

Abbreviations: ART, antiretroviral therapy; CI, confidence interval

Table S12. Weighted prevalence of each level of the HIV care cascade and cumulative incidence of mortality by end of the calendar year, using an adherence level of 80% and using September 30, 2015 as the administrative censoring date for all states

Calendar Year	Not retained Not ART adherent		Retained Not ART adherent		Not retained ART adherent		Retained ART adherent		Mortality Incidence	
	%	95% CI	%	95% CI	%	95% CI	%	95% CI	%	95% CI
2002	25.0	(24.6, 25.3)	43.2	(42.8, 43.5)	5.6	(5.4, 5.8)	22.0	(21.7, 22.3)	4.3	(4.1, 4.4)
2003	25.7	(25.4, 26.1)	37.9	(37.6, 38.2)	6.0	(5.8, 6.1)	23.7	(23.4, 24.1)	6.6	(6.5, 6.8)
2004	24.6	(24.2, 24.9)	35.0	(34.6, 35.3)	7.3	(7.1, 7.5)	24.2	(23.8, 24.5)	9.0	(8.8, 9.3)
2005	24.7	(24.4, 25.1)	32.7	(32.3, 33.1)	7.3	(7.1, 7.5)	24.0	(23.7, 24.4)	11.3	(11.1, 11.6)
2006	22.9	(22.6, 23.2)	28.2	(27.9, 28.6)	8.2	(8.1, 8.4)	27.2	(26.9, 27.5)	13.5	(13.2, 13.7)
2007	23.4	(23.1, 23.8)	25.1	(24.7, 25.4)	8.9	(8.7, 9.1)	27.4	(27.0, 27.7)	15.2	(14.9, 15.5)
2008	19.6	(19.2, 20.0)	25.4	(25.1, 25.8)	7.7	(7.5, 7.9)	30.2	(29.8, 30.6)	17.0	(16.7, 17.4)
2009	17.2	(16.8, 17.5)	24.4	(24.1, 24.8)	6.9	(6.7, 7.0)	32.7	(32.3, 33.0)	18.8	(18.5, 19.2)
2010	17.3	(17.0, 17.6)	22.4	(22.1, 22.7)	6.0	(5.8, 6.2)	33.7	(33.3, 34.0)	20.7	(20.4, 21.0)
2011	13.3	(13.0, 13.6)	24.9	(24.6, 25.3)	5.7	(5.5, 6.0)	33.9	(33.5, 34.2)	22.2	(21.9, 22.5)
2012	13.2	(12.9, 13.5)	23.9	(23.5, 24.2)	5.4	(5.2, 5.6)	33.9	(33.5, 34.2)	23.6	(23.3, 23.9)
2013	10.7	(10.4, 11.0)	25.2	(24.9, 25.5)	4.6	(4.5, 4.8)	34.5	(34.1, 34.8)	25.0	(24.7, 25.3)
2014	11.7	(11.4, 11.9)	27.4	(27.1, 27.7)	4.5	(4.4, 4.7)	30.5	(30.2, 30.8)	25.9	(25.6, 26.3)
2015	14.1	(13.9, 14.3)	27.6	(27.4, 27.9)	6.8	(6.6, 6.9)	25.0	(24.8, 25.3)	26.5	(26.1, 26.8)

Abbreviations: ART, antiretroviral therapy; CI, confidence interval
